# Microarray Technology for the Diagnosis of Fetal Chromosomal Aberrations: Which Platform Should We Use?

**DOI:** 10.3390/jcm3020663

**Published:** 2014-06-20

**Authors:** Evangelia Karampetsou, Deborah Morrogh, Lyn Chitty

**Affiliations:** 1 NE Thames Regional Genetics Service, Great Ormond Street Hospital for Children NHS Foundation Trust, London, 37 Queen Square, London WC1N 3BH, UK; E-Mails: evangelia.karampetsou@gosh.nhs.uk (E.K.); deborah.morrogh@gosh.nhs.uk (D.M.); 2 UCL Institute of Child Health, 30 Guilford Street, London WC1N 1EH, UK; 3 University College Hospital NHS Foundation Trust, London NW1 2PG, UK; 4 Great Ormond Street Hospital for Children NHS Foundation Trust, London WC1N 3JH, UK

**Keywords:** prenatal microarray, implementation, BAC, SNP

## Abstract

The advantage of microarray (array) over conventional karyotype for the diagnosis of fetal pathogenic chromosomal anomalies has prompted the use of microarrays in prenatal diagnostics. In this review we compare the performance of different array platforms (BAC, oligonucleotide CGH, SNP) and designs (targeted, whole genome, whole genome, and targeted, custom) and discuss their advantages and disadvantages in relation to prenatal testing. We also discuss the factors to consider when implementing a microarray testing service for the diagnosis of fetal chromosomal aberrations.

## 1. Introduction

In the last five years, genomic microarrays (arrays) have been widely used diagnostically for postnatal cases with developmental delay (DD), autism and/or congenital abnormalities. The increased diagnostic yield of 15%–20%, when compared to conventional karyotyping, prompted the recommendation by an international expert panel that microarray should become the first line test for postnatal referrals with these indications [1].

However, introduction of microarray testing for prenatal cases has been slow, mainly due to the potential difficulties interpreting copy number variations (CNV) of unknown significance and incomplete penetrance or variable expressivity, in the absence of a clear phenotype. Further counselling challenges arise from the coincidental discovery of late-onset disorders, which may also reveal a potentially-affected parent. In addition, obtaining sufficient DNA and good quality microarray results from prenatal samples can be difficult. 

Several recent large prospective studies have demonstrated the feasibility and utility of microarray in the prenatal setting, showing an increased diagnostic yield over karyotype for all indications for testing and in particular for referrals with sonographic abnormalities [2,3,4]. Comparison of abnormality rates among studies is difficult, and not necessarily meaningful, due to the diverse study designs, variation in microarray platforms used and inclusion criteria applied. Nevertheless, a meta-analysis of studies has shown a diagnostic yield of around 2.4% over karyotype for any referral reason and a diagnostic yield of 7% over karyotype for cases with abnormal ultrasound scan findings [5,6].

In light of these observations in December 2013 the American College of Obstetricians and Gynecologists (ACOG) issued a statement recommending the use of microarray as the first-line test for prenatal cases with structural abnormalities, fetal demise, or stillbirth. In the absence of sonographic findings the recommendation was that either karyotype or microarray could be used [7].

**Figure 1:**
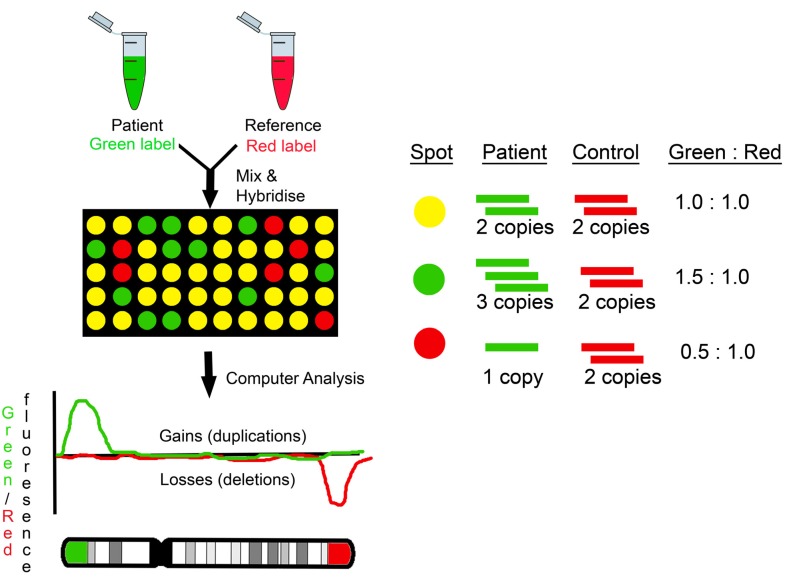
Comparative Genomic Hybridisation (CGH) arrays.

**Figure 2:**
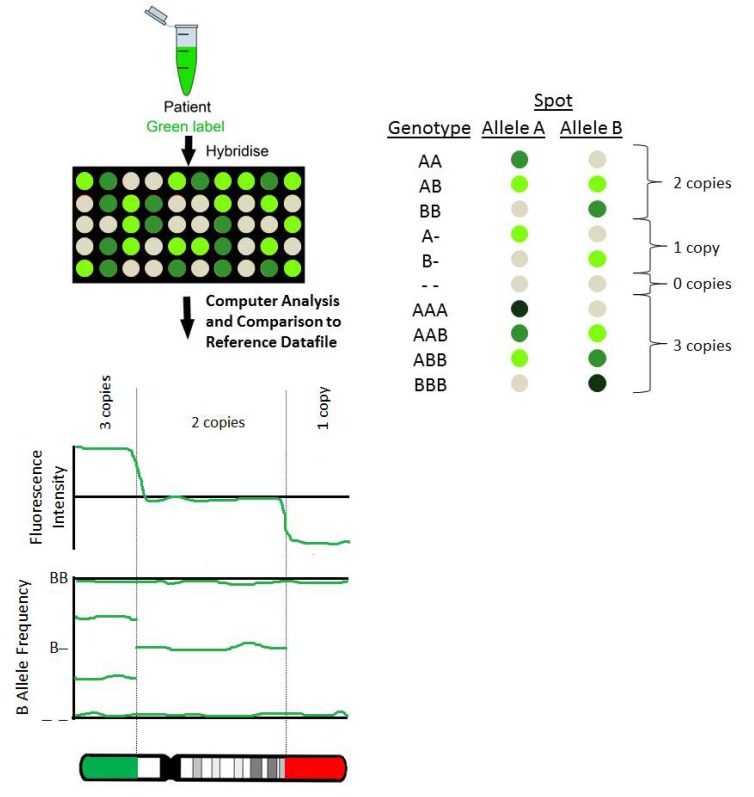
Single Nucleotide Polymorphism (SNP) arrays.

Genomic microarrays were initially developed using Bacterial Artificial Chromosome (BAC) clones. Subsequently, oligonucleotide (oligo) microarrays were developed and are now widely used worldwide in diagnostic laboratories. Oligo arrays can be either Comparative Genomic Hybridisation (CGH) or Single Nucleotide Polymorphism (SNP) arrays. Both the BAC arrays and oligo CGH arrays are based on CGH technology (Figure 1). SNP array technology is based on the discrimination between the two possible SNP alleles (A or B) for a specific position in the genome (Figure 2). For comprehensive reviews on the microarray technologies used by various manufacturers refer to Carter [8] and Alkan *et al.* [9].

Figure 1: The patient DNA and a normal control DNA, used as reference are differentially labelled using fluorescent dyes. The two DNAs are then mixed together and hybridised on a microarray slide bearing immobilised BAC (Bacterial Artificial Chromosome) or oligo (oligonucleotide) probes. Each probe, *i.e.*, each spot on the slide, represents a specific locus in the genome. The DNAs will bind to probes with complementary sequence. After hybridisation the slide is scanned and the fluorescence of each dye for each probe measured. The relative intensity between the two fluorescent dyes is calculated for each probe. The normal copy number for any locus in the genome is usually 2. An equal intensity (sample:control 2:2) for the two dyes, would represent the same amount of sample (two copies) and control (two copies) DNA and therefore a normal copy number. Higher intensity for the sample DNA (sample:control 3:2) would represent a gain (three copies), while a lower intensity for the sample DNA (sample:control 1:2) would represent a loss (one copy) for the particular probe. Analysis software plots each probe along the length of the chromosomes depending on its location and also on, above or below a baseline (that represents the normal copy number) according to its relative fluorescent dye intensity. 

Figure 2: The patient DNA is labelled using a fluorescent dye and is subsequently hybridised on a microarray slide. Each spot on the slide represents either the A or B allele for a specific locus in the genome. Both alleles are represented multiple times on the array. The DNA will bind to probes with complementary sequence. After hybridisation the slide is scanned by a scanner that measures the fluorescence of each probe. The fluorescence intensity for each locus and for each allele at that locus is calculated. A heterozygous locus (AB) will show equal intensity for both alleles. A homozygous locus (AA or BB) will show higher intensity for the allele present (A or B). Copy number information can also be extrapolated from this data. Analysis software compares the data to a reference datafile *in silico* and plots each probe along the length of the chromosomes depending on its location and fluorescence intensity, but also on the B Allele Frequency (BAF) plot according to the presence or absence and fluorescent intensities of the A and B alleles.

Here, we compare the performance of different array platforms and designs with regards to appropriateness for use for prenatal testing (Table 1). We will also discuss the factors to consider when implementing a microarray testing service for the diagnosis of fetal chromosomal aberrations.

## 2. Types of Array Platforms

### 2.1. BAC Arrays

The first microarray platforms utilised BAC clones derived from the Human Genome Project. In more recent years they have been largely replaced by oligo-based platforms, either CGH or SNP, due to the higher resolution that these platforms can offer. However, certain features of BAC microarrays make them potentially appealing in a prenatal setting.

**Table 1:** Comparison between Karyotype, Bacterial Artificial Chromosome (BAC) arrays, oligo Comparative Genomic Hybridisation (CGH) and Single Nucleotide Polymorphism (SNP) arrays.

			Array Platform	
	Karyotype	BAC	Oligo CGH	SNP
Resolution	5–10 Mb	0.5–1 Mb	0.05–0.4 Mb	0.05–0.4 Mb
			depending on specific platform, design and calling settings	
Diagnostic yield (excluding common aneuploidies)	around 5%	higher than karyotype	higher than BAC arrays	higher than BAC arrays
		Diagnostic yield almost double compared to karyotype		
Detection of CNVs of unknown significance	+	+	++	++
Detection of CNVs of reduced penetrance and variable expressivity	−	+	+	+
Starting material (ng)		50	1000 (200–2000)	200–250
Turnaround time (working days) (+ 1 day if a rapid result is needed in advance)	6–10	3	4	4–7
Multiplexing/Throughput	−	2 samples per slide	1–8 samples per slide multiplexing in 94-well plates possible	1 sample per chip or 8 samples per slide multiplexing in 94-well plates possible
Detection of MCC	possible only if the fetus is male	−	−	+
Detection of triploidy	+	−	−	+
Detection of LOH/UPIC	−	−	−	+
Detection of mosaicism	+	Depends on size of the locus, type of aberration, platform, normalisation and calling algorithms. Possibly easier detection using SNP arrays		
Cost	comparable to microarray	Depends on throughput, specific platform and overhead costs		

BAC = Bacterial Artificial Chromosome, Oligo = Oligonucleotide, CGH = Comparative Genomic Hybridisation, SNP = Single Nucleotide Polymorphism, CNV = Copy Number Variation, MCC = Maternal Cell Contamination, LOH = Loss of Heterozygosity, UPID = UniParental IsoDisomy.

Diagnostic laboratories usually receive Chorionic Villus Samples (CVS) or Amniotic Fluid (AF). Given that the amount of material is often limited and back-up cultures need to be set up, only a minimal amount of CVS or AF is available for DNA extraction. It can be challenging to obtain DNA of adequate quantity and quality for microarray testing, but this problem can be resolved when using a BAC platform, since the recommended starting material is only 50 ng, an amount easily obtainable from most samples.

BAC clones are 100–150 kb long and therefore generate an intense hybridisation signal with a high signal-to-noise ratio. This translates to a good microarray profile and a robust, reproducible assay, making them an attractive choice for the often poor quality prenatal DNA.

DNA could, of course, be extracted from cultured cells instead of direct CVS or AF samples, providing enough material for high quantity and quality DNA extraction. This would, however, add considerably to the reporting time and is to be avoided for prenatal samples where time is limited. In addition, cultural artefacts can arise that may require extra culturing and karyotyping work to determine their significance.

BAC arrays have an average whole genome resolution of 0.5–1 Mb, which is around 10 times higher than conventional karyotyping, but is lower than oligo-based arrays. However, this could be considered desirable in a prenatal setting, because although increasing resolution increases the diagnostic yield in postnatal cases [10], it also increases the detection of CNVs of unknown significance [11,12]. Abnormal sonographic findings are often non-specific making clinical interpretation of microarray findings of unknown significance challenging. From the laboratory perspective, interpretation can be difficult, time-consuming and potentially inconclusive, even when parental samples are available and inheritance studies are undertaken. More importantly, from the clinical and patient perspective, such microarray findings can lead to difficult counselling creating a lot of uncertainty and anxiety to the parents and potentially leading to the termination of healthy wanted pregnancies.

The first BAC arrays required a dye-swap experiment. The DNA of interest and normal reference control DNA were labelled twice each, swapping fluorescent dyes, and hybridised on two sub-arrays (hybridisation areas) on a microarray slide. This resulted in one sample being hybridised per slide. Newer BAC platforms, protocols and analysis software have been developed, so that dye-swap is no longer necessary. This allows two samples to be set up per array slide. Even so, multiplexing is limited and BAC arrays are only suitable for low throughput. In a laboratory receiving many prenatal samples, this would be labour intensive and would not be cost-effective. In addition, it is now common practice to use higher resolution platforms for postnatal referrals, leading to the requirement for two different platforms, one for postnatal and another for prenatal testing, and the concomitant logistical difficulties this may entail. However, in a smaller laboratory that receives only a few samples a week, a BAC platform may be cost-effective and could minimise any delay in reporting as only two samples are required per run. BAC arrays protocols are 1–1.5 day long, giving the potential of a very fast result. Theoretically, from sample receipt to reporting it could take less than three days.

To our knowledge, currently there is only one available off-the-shelf BAC platform on the market. This is largely for users of prenatal arrays, but it means there are very limited options regarding array design and supplier.

### 2.2. OligoCGH Arrays

OligoCGH arrays are widely used in the postnatal setting. Oligo probes are ~50–60 bp long (2000–2500 times shorter than BAC probes), and thus have the potential to provide higher resolution, which is dependent on the number and spacing (density) of the probes, as well as on the number of consecutive probes needed for a CNV call to be made. Whereas BAC arrays have a high signal-to-noise ratio and therefore a single BAC clone is sufficient for accurate CNV calling, shorter oligo probes result in less specific hybridisation and lower signal-to-noise ratio, *i.e.*, higher background noise. For oligo-based arrays this leads to less robust assays and higher number of consecutive probes required for a call to be made confidently. This means that the resolution can be varied depending on the platform and the calling parameter settings adopted after the validation of the platform. A minimum resolution of 400 kb has been recommended [13].

Higher resolution results in the detection of smaller CNVs. Therefore, as already mentioned, the diagnostic yield is higher when using an oligo-based (either CGH or SNP) compared to BAC arrays [10,14]. With higher diagnostic yield, usually comes a higher rate of CNVs of unknown significance [11,12]. However, for a variety of reasons, the final detection rate of changes of unknown significance is similar for BAC and oligoCGH arrays, although the nature of uncertainty is different. Both platforms can lead to uncertainty because of limited or absent data in in-house and publically available databases relevant to the CNV detected and the genes within it. This type of uncertainty is higher in oligo arrays. In contrast, when using BAC arrays, interpretational difficulties arise because of the large intervals between consecutive probes and therefore uncertainty as to the gene content [14]. Oligo-based arrays provide better description of the breakpoints and therefore, more precise delineation of the genes involved. Such CNVs are often inherited, making interpretation easier in light of the parent’s phenotype. *De novo* CNVs are less frequent and could be causative of an abnormal phenotype. In addition, although such “private” CNVs of unknown significance might be more frequently encountered in oligo-based platforms, recurrent CNVs of variable expressivity or incomplete penetrance, such as the reciprocal duplication of the DiGeorge Syndrome region (dup(22)(q11.2q11.2)), are equally prevalent in both BAC and oligo-based arrays, posing the same difficulties in interpretation, reporting, and counselling.

Microarrays have now been in clinical use for a few years, and online databases with results from normal individuals (such as the Database of Genomic Variation [15]), and those with phenotypic abnormalities (such as DECIPHER [16] and ECARUCA [17]), are widely available. In addition, databases cataloguing genes (Online Mendelian Inheritance in Man (OMIM)) and genomic regions (DECIPHER) that cause disease are available. The International Standards for Cytogenomics Array (ISCA) consortium provide regular reviews and updates on haplo-insufficiency or triplo-sensitivity scores assigned to genes [18]. These databases are indispensable tools that aid the interpretation of both postnatal and prenatal microarrays, minimising uncertainty in reporting. Additionally, as datasets of both normal control subjects and subjects being tested by microarray are becoming larger, the prevalence of relatively rare recurrent CNVs of variable expressivity or incomplete penetrance can be more accurately calculated. Over time this improves our understanding of CNVs and helps in the counselling of parents when the fetus carries such a CNV [19,20].

The resolution of the array used and the reporting of CNVs of unknown significance vary in different parts of the world [11]. This is reflected by the fact that the majority of laboratories in the USA use the highest resolution arrays, while in most European countries laboratories use lower resolution oligo-based arrays.

The amount of DNA available from prenatal samples can be limited, and the recommended starting material varies between different platforms from as little as 200 ng to 2000 ng of DNA. An average would be 1000 ng, but practical experience in our laboratory has shown that even with half the amount of DNA (500 ng), good array results with quality and profiles comparable to postnatal results can be consistently obtained from prenatal samples. Laboratories should validate their platform for lower DNA starting material, in order to maximise its utility for prenatal samples with low quantities of DNA.

There are a variety of commercially available oligoCGH platforms. Depending on the probe density used, these platforms can be used to multiplex between one and eight samples per slide. Higher multiplexing results in streamlined workflow, lower array price per sample, improved turnaround times and overall cost-efficiency. Moreover, protocols which adopt multiplexing the labelling process in 94-well plates can further increase the throughput, rendering these platforms appropriate for laboratories that receive large number of samples per week. OligoCGH array protocols are 1.5–2 days long, adding potentially 0.5–1 day of turnaround time compared to BAC arrays. However, results could still be reported within four days from sample receipt.

### 2.3. SNP Arrays

SNP arrays were initially designed for and used in studies examining the association of specific SNPs with disease (Whole-Genome Association Studies). Later, their use was expanded to the detection of CNVs.

SNP arrays use oligo probes that are either 25 bp or 50 bp long, and therefore tend to have the lowest signal-to-noise ratio compared to other platforms. Again, as with oligoCGH arrays, resolution will depend on the probe density and the calling algorithm. SNP arrays can be designed with different numbers of SNPs across different parts of the chromosome and resolution will be higher in targeted areas. SNP arrays tend to have the highest probe density compared to BAC and oligoCGH arrays, and therefore they allow more accurate delineation of the breakpoints and the genes, or even the introns and exons of a specific gene involved. Despite this, as with oligoCGH, interpretational difficulties might arise when private CNVs are encountered but in-house and online available databases, as well as inheritance studies, will resolve the vast majority of these.

The starting material recommended is between 200 ng and 250 ng of DNA, which is in general an achievable target for the majority of prenatal samples. As with other array platforms, even lower amounts of starting material are likely to give microarray results of adequate quality for analysis. As with oligo arrays, there are a variety of platforms available, many of which can be used to multiplex samples in batches of up to 24. Protocols vary and can last three to five days, adding considerable time to the results turnaround time. Nevertheless, results can be reported within five to seven days from sample receipt, which is usually an acceptable timeframe for prenatal samples, particularly when compared to karyotyping, where average reporting times are between 10–14 days.

The disadvantage of SNP arrays results from the fact that probe spacing and genome coverage is limited by the non-random distribution of SNPs in the genome. Regions of segmental duplications are usually poorly or not at all covered. SNP array manufactures now provide arrays that also have non-polymorphic SNP-independent copy number probes providing more even and consistent coverage.

The major advantage of SNP arrays over other platforms, and of particular relevance to prenatal diagnosis, is the additional data gained by the SNP probes. This data, presented usually as B Allele Frequency (BAF), provides valuable genotyping information by producing specific BAF patterns in cases of triploidy, diploid-triploid mosaicism, maternal cell contamination (MCC), and loss of heterozygosity (LOH). These patterns render such cases readily detectable. Triploidy and diploidy-triploidy mosaicism are relatively frequent in prenatal diagnosis (1%–3% of conceptions) and would not be detected by BAC or oligo arrays. MCC is thought to occur in around 2.5% of cases, especially in bloodstained amniotic fluid samples [21]. A SNP array would detect the presence of the maternal genotype in the sample and indicate that the DNA tested is not purely fetal, thereby decreasing the reliability of the results. The presence of LOH could be either due to common ancestry of the parents (consanguinity) or due to UniParental IsoDisomy (UPID). UPID is clinically relevant for certain imprinted regions of the genome (e.g., 15q11q13 is associated with Prader-Willi and Angelman Syndromes). However, the diagnostic yield for clinically relevant UPID is very low because the incidence of uniparental disomy is rare [22]. Alternatively, LOH for a whole acrocentric chromosome might represent an isochromosome, which would alert the laboratory to further investigate the fetus and parents by karyotyping to assess future reproductive risks [23].

One further advantage is that the genotype information provided by SNP arrays allows more accurate copy number determination than CGH (BAC or oligo) arrays because the latter delivers a relative quantitation technique, where gains and losses in comparison to the reference normal genome are detected but the exact copy number cannot be easily extrapolated. The exact copy number is more easily extrapolated by the BAF plot generated from SNP array analysis, which allows homozygous and heterozygous deletions to be easily distinguished. Similarly, three copies can be easily distinguished from four copies at a specific locus, which might indicate the specific underlying mechanisms of chromosomal aberration formation. In general, the SNP data provides confirmation of the copy number data, minimising technical artefacts and false-positive calls.

It has also been suggested that mosaicism is more readily detectable by SNP arrays, due to the presence of the SNP data. However, direct comparisons among platforms are difficult, because the lowest level of mosaicism detected will not only depend on the size of the locus and type of aberration, but also on the microarray data normalisation and calling algorithms [13].

Finally, when using CGH platforms the patient and control DNA need to be sex-matched and therefore the fetal sex needs to be known before the microarray testing. The technology of SNP arrays is not based on CGH, as only the patient DNA is hybridised on the microarray slide or chip. The data produced is then compared to a reference data-file, containing the results from multiple controls. As knowledge of fetal sex is not required before SNP analysis, no rapid sex determination using QF-PCR (Quantitative Fluorescent—Polymerase Chain Reaction) is required. This offers a further advantage over oligo arrays, where this step must be undertaken before analysis.

## 3. Array Design

### 3.1. Targeted

In order to minimise findings of unknown clinical significance, some of the first BAC platforms designed were targeted, only covering regions of the genome linked to known syndromes, as well as subtelomeric and pericentromeric regions. Backbone probes, *i.e.*, probes evenly spaced across the genome providing low whole genome coverage, were not included in these designs and, therefore, such platforms showed large coverage gaps. The majority of pathogenic CNVs are non-recurrent; thus, pathogenic abnormalities may be missed, while the incidence of CNVs of unknown significance is not necessarily decreased [24].

Targeted designs are not commercially available currently and, if a laboratory wished to use such a platform, it would have to be custom-made. The authors’ opinion is that targeted designs should be avoided, due to the significant likelihood of even large clinically relevant CNVs being missed. A design with at least a low-resolution backbone would be more appropriate in the prenatal setting.

### 3.2. Whole-Genome

As the name implies, whole-genome designs include probes covering the whole genome. Probes are usually spaced in more or less equal intervals, which overcome the problem of missing large clinically-relevant CNVs encountered in targeted designs.

Whole-genome designs tend to produce array profiles of good quality with few, if any, false-positive calls. However, the vast majority of commercially available designs are whole-genome and targeted.

### 3.3. Whole-Genome and Targeted

Most commercially available designs follow the whole-genome and targeted format. A probe backbone covering the whole genome is present. Moreover, additional probes are included, targeting regions and genes of clinical interest. Regions and genes of interest can be identified from resources such as peer-reviewed publications and OMIM. The most commonly adopted list of targeted genes and regions is the ISCA design, which is based on the haplo-insufficiency and triplo-sensitivity scores assigned by the ISCA consortium [18].

The main drawback of targeting specific loci is that the design needs to be constantly under review and frequently updated in line with the new evidence published. For instance, although recent evidence clearly supports the fact that haplo-insufficiency of the gene *ARID1B* leads to intellectual disability [25,26] and the gene has been assigned the highest haplo-insufficiency score by the ISCA consortium, the gene is not targeted in the current version 2 ISCA microarray design, because it was defined since the last update. Similarly, genes that are targeted might be under review and may not be targeted in future versions.

Furthermore, because of the targeted nature of the design, probe coverage is not uniform across the genome. In our experience, labelling issues or relatively poor quality array profiles could lead to false-positive CNVs of whole genes that are targeted in these designs. Such artefacts are much less frequent in whole-genome non-targeted designs. Therefore, microarray analysts should be aware of such artefacts and vigilant when interpreting targeted single gene gains or losses. Microarray quality metrics should always be taken into consideration.

### 3.4. Custom Designs

Laboratories have the option of designing their own array platforms, whether targeted, whole-genome or both. Again, as with off-the-shelf platforms, targeted regions and genes should be frequently reviewed and the design should be updated accordingly. Furthermore, since the design and probes included will not have been validated by the array supplier, additional validation locally might be necessary.

## 4. Other Factors to Consider When Implementing a Prenatal Array Service

### 4.1. Experience

One of the most important factors to consider before offering a prenatal microarray service is the individual laboratory’s experience of arrays [27]. The previous and current experience of specific platforms is an invaluable tool in the successful implementation of prenatal arrays. As mentioned above, in-house databases will greatly aid interpretation, even if they are mainly built on results from postnatal samples. Moreover, practical experience of the array protocol and behaviour of the specific platform will help implement a prenatal service by simplifying the validation process and aiding troubleshooting. We consider the experience in postnatal samples not only desirable, but also an indispensable component for the implementation of microarrays in a prenatal setting. We recommend that the same platform should be used for both postnatal and prenatal referrals. In addition to interpretation, this will assist in and simplify the laboratory’s workflow, saving considerable labour time. Laboratories that only offer a prenatal service should not be discouraged from implementing microarrays. However, they will need to choose an array platform and design that they feel confident analysing and undertake extensive validation.

### 4.2. Costs

Although the features of different platforms and designs described here are of utmost importance when choosing a prenatal microarray, the cost of implementation cannot be ignored. Cost per patient can vary between different platforms and suppliers. For the same type of platform, cost increases with increasing resolution. Additionally, if the microarray service is being introduced in the laboratory, further setting-up costs will apply and they may vary widely. In general, reagent costs tend to be comparable for platforms of similar resolution: Therefore, set-up, validation, and the ability to multiplex, and thereby increase efficiency, may be deciding factors when selecting an array platform. In addition to reagent and hardware expenses, labour may add considerably to the cost. Labour cost varies across the world and high-cost labour could be offset by automation and fast, intuitive, user-friendly analysis software.

### 4.3. Analysis and Analysis Software

As already mentioned, it is crucial for the analysis software to be fast, intuitive and user-friendly, not only in order to reduce analysis time and labour costs, but also to aid with interpretation. The analysis software should have links to all the major genome browsers and variant databases (such as DGV, DECIPHER, ECARUCA, ISCA, OMIM). If this data is embedded in the software, instead of being real-time, then frequent software updates should be released by the software supplier. The analysis software should also have the capacity of creating and storing a database with all the previous laboratory microarray results and their interpretation. This in-house database would be of utmost importance during analysis of future cases, especially since some CNVs can be platform-specific benign variants or artefacts.

Most array platforms come with complimentary analysis software but this should be assessed and deemed fast and intuitive by the user. If the software is not user-friendly, then purchasing a license for alternative software should be considered, especially if this would save considerable analysis and interpretation time. In addition, different software programmes utilise different algorithms and thus their performance can be very variable [28,29] and can affect the detection of CNVs.

In order to minimise the detection of CNVs of unknown significance, some laboratories may choose to filter the microarray results and apply CNV size cut-off filters. However, the gene content is more informative regarding interpretation than the size of the CNV. Therefore, in our view, even when using a filter, CNVs of unknown significance, variable expressivity or incomplete penetrance, will still be detected with significant chromosomal abnormalities being missed.

## 5. Conclusions

The advantage of microarray over conventional karyotype for the diagnosis of fetal pathogenic chromosomal aberrations has been proven [2,3,4] and is no longer debatable [5,6]. However, this increased yield of abnormal chromosomal abnormalities brings with it counselling challenges subsequent to the interpretational difficulties associated with the increased detection of CNVs of variable or unknown significance.

There are several array platforms and designs available, each with their own advantages and disadvantages. Many factors will impact on the decision as to which to use (Table 2) and will be a balance between the diagnostic yield, detection of CNVs of unknown significance and implementation costs. Regardless of which platform is used, awareness by the laboratory, as well as by the clinicians and genetic counsellors, of the limitations and advantages of the platform is crucial, in order to limit unrealistic expectations and facilitate appropriate pre-test and post-test counselling.

**Table 2:** Choosing a microarray platform checklist.

1	Current platform used by the laboratory; incorporation in current workflow
2	Existing experience with and results in-house database from current/previous platforms
3	Platform resolution; diagnostic yield and potential detection of CNVs of unknown significance
4	Platform design; whole-genome/targeted, off-the-self/custom-made
5	Cost for setting-up a prenatal microarray service
6	Reagents cost per patient; potential for multiplexing
7	Starting material; DNA extraction method and DNA yield
8	Report turnaround time
9	SNP information; detection of triploidy/MCC/LOH
10	Analysis software

In our era of rapidly developing genetic technologies, it is to be anticipated that next generation sequencing (NGS) will replace array technology both postnatally and prenatally. The use of non-invasive prenatal testing (NIPT) for aneuploidy by analysis of cell free fetal DNA in maternal blood is now a reality [30] and it is expected to dramatically reduce the number of invasive procedures and tests [31]. As the NIPT technology becomes more robust and affordable, it will also have the potential to be used for the detection of fetal CNVs [32] and eventually fetal whole genome sequencing [33], potentially replacing invasive testing and prenatal microarrays altogether.
